# Identification of putative pathogenic single nucleotide variants (SNVs) in genes associated with heart disease in 290 cases of stillbirth

**DOI:** 10.1371/journal.pone.0210017

**Published:** 2019-01-07

**Authors:** Ellika Sahlin, Anna Gréen, Peter Gustavsson, Agne Liedén, Magnus Nordenskjöld, Nikos Papadogiannakis, Karin Pettersson, Daniel Nilsson, Jon Jonasson, Erik Iwarsson

**Affiliations:** 1 Department of Molecular Medicine and Surgery and Center for Molecular Medicine, Karolinska Institutet and Karolinska University Hospital, Stockholm, Sweden; 2 Department of Clinical Pathology and Clinical Genetics, and Department of Clinical and Experimental Medicine, Linköping University, Linköping, Sweden; 3 Center for Perinatal Pathology and Department of Pathology, Karolinska University Hospital, Huddinge and Karolinska Institutet, Stockholm, Sweden; 4 Department of Obstetrics and Gynecology, Karolinska University Hospital, Huddinge and Karolinska Institutet, Stockholm, Sweden; Pennsylvania State University, UNITED STATES

## Abstract

The incidence of stillbirth in Sweden has essentially remained constant since the 1980’s, and despite thorough investigation, many cases remain unexplained. It has been suggested that a proportion of stillbirth cases is caused by heart disease, mainly channelopathies. The aim of this study was to analyze DNA from 290 stillbirth cases without chromosomal abnormalities for pathogenic single nucleotide variants (SNVs) in 70 genes associated with cardiac channelopathies and cardiomyopathies. The HaloPlex Target Enrichment System (Agilent Technologies) was utilized to prepare sequencing libraries which were sequenced on the Illumina NextSeq platform. We found that 12.1% of the 290 investigated stillbirth cases had one (n = 31) or two (n = 4) variants with evidence supporting pathogenicity, *i*.*e*. loss-of-function variants (nonsense, frameshift, splice site substitutions), evidence from functional studies, or previous identification of the variants in affected individuals. Regarding identified putative pathogenic variants in genes associated with channelopathies, the prevalence was significantly higher in the stillbirth cohort (n = 23, 7.93%) than the corresponding prevalence of the same variants in the non-Finnish European population of the Exome Aggregation Consortium (2.70%, p<0.001) and SweGen, (2.30%, p<0.001). Our results give further support to the hypothesis that cardiac channelopathies might contribute to stillbirth. Screening for pathogenic SNVs in genes associated with heart disease might be a valuable complement for stillbirth cases where today’s conventional investigation does not reveal the underlying cause of fetal demise.

## Introduction

The incidence of stillbirth in Sweden, defined as fetal death occurring at completed gestational week 22 or later, has essentially remained constant at approximately 3–4 per 1 000 live births since the 1980’s [1]. Stillbirth can be caused by several factors, such as infections, placental insufficiency or abruption, maternal conditions (*e*.*g*. preeclampsia), chromosomal aberrations, malformations and umbilical cord complications [2]. In Stockholm County, all cases of stillbirth pass a thorough investigation, with the aim of identifying the underlying factor of fetal demise. The investigation includes physical examination and autopsy, infectious disease testing and chromosome analysis by conventional chromosome analysis by karyotyping, or, when it fails, by quantitative fluorescence polymerase chain reaction (QF-PCR). Using these methods, chromosomal abnormalities are identified in 6–17% of stillbirth cases [3,4]. We have previously shown that analysis with chromosomal microarray (CMA) increases both the analysis success rate, as well as the chromosomal aberration detection frequency, compared with conventional karyotyping [5]. However, many stillbirth cases remain unexplained, and determination of the underlying cause is important as a history of stillbirth is associated with an increased recurrence risk in following pregnancies [6].

Studies have suggested that long QT syndrome (LQTS) might contribute to stillbirth in some cases [7,8]. LQTS is a channelopathy affecting cardiac ion channels, and is characterized by a prolonged Q-T interval on electrocardiogram. The condition is a common cause of sudden death postnatally, and is diagnosed in up to 9.5% of infant death syndrome cases [9]. Also other channelopathies, such as Brugada syndrome (BrS) and catecholaminergic polymorphic ventricular tachycardia (CPVT), as well as cardiomyopathies, such as hypertrophic cardiomyopathy (HCM), have been suggested to cause infant death [10–13]. Common for cardiac channelopathies is that the structure and function of ion channels are affected, which in turn leads to disrupted action potential propagation and thereby causes development of arrhythmias [14]. Cardiomyopathies, *i*.*e*. disorders of the heart muscle, are impairments of the ability of the myocardium to contract, which can result in heart failure [15]. A study including 47 cases of sudden unexpected death in infancy (SUDI) identified one or more genetic variants with likely functional effects in 34% of the cases, by investigation of 100 genes associated with cardiac channelopathies and cardiomyopathies [16]. It is reasonable to suspect that genetic variants associated with death in infancy might as well cause fetal death. However, this has not been extensively studied.

In this study, DNA from 290 stillbirth cases without chromosomal abnormalities was analyzed using a gene panel, including 70 genes associated with cardiac channelopathies and cardiomyopathies. We suggest that the results might provide clues to the underlying cause of stillbirth for a proportion of cases.

## Materials and methods

### Study population

The study included 290 stillbirth cases without known pathogenic chromosomal abnormalities, based on data from karyotyping (174 cases), CMA (84 cases), and QF-PCR (32 cases). All cases are part of a cohort that we have described previously [5], which includes all stillbirth cases that occurred in the Stockholm County between January 1, 2008 and December 31, 2012. The complete cohort has been investigated by the Stockholm stillbirth group, which consists of obstetricians and perinatal pathologists representing all delivery departments in Stockholm, according to guidelines described in the Stockholm classification of stillbirth [17]. Parental samples were not available. All data were fully anonymized prior to analysis. The study was approved by the Regional Ethical Review Board in Stockholm.

### DNA extraction, quantification, and quality control

DNA was extracted from fetal or placental tissue, using the Gentra PureGene protocol (Qiagen, Hilden, Germany). DNA concentrations were measured using a Qubit fluorometer together with the DS BR DNA assay (Thermo Fisher Scientific, Waltham, MA, USA). DNA quality was assessed using a NanoDrop spectrophotometer (Thermo Fisher Scientific), where A260/A280 ratios between 1.8 and 2.0, and A260/A230 ratios >1.5 were accepted. DNA fragmentation was evaluated using agarose gel electrophoresis (1.5% agarose).

### Panel design and library preparation

By using the SureDesign tool (Agilent Technologies, Santa Clara, CA, USA), probes were designed to cover the exons and exon-intron boundaries of the 70 genes displayed in Table 1. The genes were selected according to the available literature and were reported to be associated with cardiac channelopathies and cardiomyopathies. The design was optimized twice through addition of probes in areas where coverage was low, both in areas with no *in silico* coverage and where actual sequencing coverage was below 20X, as well as in areas where only one probe was functional, prior to running stillbirth cases. Sequencing libraries for massive parallel sequencing (MPS) were obtained by using HaloPlex target enrichment system (Agilent Technologies) according to the manufacturer’s protocol. Briefly, 225 ng of genomic DNA was used for restriction reactions. Before continuation of the protocol, the digestion of a control DNA sample was assessed using a high-sensitivity DNA kit and Bioanalyzer (Agilent Technologies), to ensure that the restriction reaction was successful and yielded DNA fragments of the expected lengths. Hybridization of the probes and index cassettes was performed at 54°C for 16 hours ± 10 minutes. PCR amplification of all libraries was performed on a 2720 Thermal cycler (Applied Biosystems, Foster City, CA, USA). The number of PCR cycles was adjusted to 19 instead of the recommended 20, as signs of overamplification were noted in the quality control of the first batch of processed samples. The last step of the protocol, *i*.*e*. bead-based purification of the libraries, was routinely done twice, as a single purification was insufficient to diminish the 125 bp peak generated by an adapter-primer complex, which is a common byproduct according to the manufacturer. The finished libraries were once again quality controlled using a high-sensitivity DNA kit and Bioanalyzer (Agilent Technologies), and quantified using a Qubit fluorometer (Thermo Fisher Scientific). Libraries were pooled to contain 30 samples for medium output sequencing reagents, or 90 samples for high output sequencing reagents. The final concentration of the pools was 4 nM, and 1 pM was used as input for sequencing. Sequencing was performed using NextSeq reagent kit version 2, 300 cycles, on the NextSeq instrument (Illumina Inc., San Diego, CA, USA). The obtained cluster densities were between 180 000–230 000 clusters/mm2.

**Table 1:** Genes included in the customized HaloPlex gene panel and their associated diseases.

Disease category	Genes
Channelopathies	*AKAP9* (1), *ANK2* (1, 2, 3), *CACNA1C* (2), *CACNA2D1* (2, 4), *CACNB2* (2), *CASQ2* (3), *GPD1L* (2, 5, 6), *HCN4* (2, 7), *KCNE1* (1, 8), *KCNE2* (1, 10), *KCNE3* (2), *KCNH2* (1, 4), *KCNJ2* (1, 8, 4), *KCNJ5* (1), *KCNJ8* (2, 6), *KCNQ1* (1, 4, 8), *RYR2* (3, 1, 9), *SCN1B* (2, 8), *SCN3B* (2, 8), *SCN4B* (2, 8), *SCN5A* (1, 2, 6, 7, 8, 10, 11, 12), *SNTA1*(1, 6, 13), *TRPM4* (2, 10)
Cardiomyopathies	*ABCC9* (12), *ACTC1* (12, 13, 14, 15), *ACTN2* (12, 14), *ANKRD1* (12), *BAG3* (12), *BRAF* (14, 15), *CALR3* (14), *CAV3* (1, 6, 14), *CSRP3* (12, 13, 14), *DES* (12), *DMD* (12), *DNAJC19* (12), *DSC2* (9), *DSG2* (9, 12), *DSP* (9, 12), DTNA (13, 15), FHL2 (12), *GLA* (14), *JPH2* (14), *JUP* (9), *LAMP2* (14), *LDB3* (9, 12, 13), *LMNA* (9, 12), *MIB1* (12), *MYBPC3* (12, 13, 14), *MYH7* (12, 13, 14), *MYL2* (14), *MYL3* (14), *MYLK2* (14), *MYOZ2* (12, 14), *NEBL* (12), *NEXN* (12, 14), *PLN* (9, 12, 14), *PRKAG2* (14), *PKP2* (9, 2), *RBM20* (12), *TAZ* (12, 13), *TCAP* (12, 14), *TGFB3* (9), *TMEM43* (9), *TMPO* (12), *TNNC1* (12, 14), *TNNI3* (12, 14), *TNNT2* (12, 13, 14), *TPM1* (12, 13, 14), *TTN* (12, 14), *VCL* (12, 14)

(1) Long QT syndrome (LQTS), (2) Brugada syndrome (BrS), (3) Catecholaminergic polymorphic ventricular tachycardia (CPVT), (4) Short QT syndrome (SQTS), (5) Right bundle branch block (RBBB), (6) Sudden infant death syndrome (SIDS), (7) Sick sinus syndrome (SSS), (8) Familial atrial fibrillation (FAF), (9) Arrhythmogenic right ventricular cardiomyopathy (ARVC), (10) Cardiac conduction disease (CCD), (11) Paroxysmal familial ventricular fibrillation (PFVF), (12) Dilated cardiomyopathy (DCM), (13) Left ventricular non-compaction (LVNC), (14) Hypertrophic cardiomyopathy (HCM), (15) Congenital heart defects (CHD)

### HaloPlex data analysis and bioinformatics

A previously described custom script pipeline [18], originally developed to achieve a faster analysis procedure, and which has been shown to yield results of higher quality than other pipelines commonly used for analysis of MiSeq HaloPlex MPS data, was modified to eliminate the detrimental effects of the higher frequency of erroneous low quality base calls obtained with the Illumina NextSeq instrument used in the present series. Instead of removing reads with low quality bases ≤Q13, the low quality base calls were N-substituted and paired-end analysis was performed (without pooling of identical read pairs) using bwa-mem 0.7.12 [19]. The sequence reads were mapped against the human genome version GRCh37/hg19. The resulting SAM-files were used for further analysis as described earlier. Integrative Genomics Viewer (IGV, Broad Institute, Cambridge, MA, USA) was used for visualization of BAM-files, and Alamut Visual (Interactive Biosoftware, Rouen, France) was used for annotation and evaluation of identified SNVs. Combined Annotation Dependent Depletion (CADD) scores were used to assess the deleteriousness of the variants [20]. In contrast to other tools used to predict functional effects of genetic variants, CADD has the advantage that it combines several different annotations to create a single score. The scaled CADD score, used in the present study, relates the variant of interest to all possible theoretical variants in the genome, and returns a logarithmic representation of the score. A score of 20 indicates that the variant is among the 1% most deleterious variants in the genome, 30 indicates that it is among the 0.1% most deleterious variants, and so on. Variants occurring at a frequency of >1% in population databases were excluded from further analysis, as were synonymous variants and intronic variants that were not predicted to affect splicing, according to the Human Splicing Finder integrated in Alamut Visual. The remaining variants were individually evaluated based on available literature, type of variant (missense, loss-of-function variant), entries in the Human Gene Mutation Database (www.hgmd.org), ClinVar (www.ncbi.nlm.nih.gov/clinvar/) and dbSNP (www.ncbi.nlm.nih.gov/SNP/), as well as in regard to evolutionary conservation and CADD score. Loss-of-function (LoF) variants were considered putative pathogenic, as were missense variants where previously published data suggested an association between the variant and the disease of interest. Variants interpreted as putative pathogenic were classified according to guidelines from the American College of Medical Genetics and Genomics (ACMG) [21]. Missense variants for which no data supporting pathogenicity had been published, and which additionally were located at poorly conserved positions and had a low CADD score, were considered likely benign and excluded. Missense variants which did not fall into either of these categories were considered to be of unknown clinical significance.

### Statistical analysis

The number of identified putative pathogenic variants in the study cohort was compared to the corresponding number for the same variants reported in the Non-Finnish European (NFE) population of the Exome Aggregation Consortium (ExAC NFE) [22] and SweGen [23], using Fisher’s exact test. Since only frequency data were available from ExAC and SweGen, this test was performed under the assumption that no individual in the different data sets was carrier of more than one putative pathogenic variant. Minor allele frequencies (MAFs) for variants identified in the study cohort were calculated and compared with the corresponding MAFs in ExAC NFE and SweGen. As the *KCNQ1* gene was the gene in which most putative pathogenic SNVs were identified (n = 5), it was selected for a gene-wide comparison of the number of putative pathogenic variants between the stillbirth cohort and ExAC NFE. All missense and LoF variants in *KCNQ1* registered in ExAC NFE were systematically searched for in ClinVar, to get an approximation of how common putative pathogenic SNVs are in the European population for this gene. The number of variants registered as “Pathogenic” or “Likely pathogenic” was compared to the number of putative pathogenic SNVs identified in the stillbirth cohort, using Fisher’s exact test.

In order to explore whether a difference in the proportion of pathogenic SNVs in the stillbirth cohort vs. ExAC NFE was present across all genes included in the HaloPlex gene panel, a Monte Carlo permutation test with 10 000 iterations was performed. Each iteration included 50 variants randomly selected from each cohort. Missense and LoF variants occurring at a frequency of <1% in ExAC NFE for the 70 genes were included, and variants reported as pathogenic or likely pathogenic was extracted from ClinVar, in order to make it comparable with the variants identified in the stillbirth cohort. For each iteration, the number of pathogenic variants was counted, whereupon the average proportion of putative pathogenic SNVs across all iterations was calculated for both cohorts. The chi^2^ test was used to assess differences in distribution of gestational age intervals of stillbirth cases harboring a putative pathogenic SNV compared with the complete cohort, whereas Fisher’s exact test was used to assess differences in sex distribution. The significance level of the analyses was set to p = 0.05.

## Results

The mean sequencing coverage for the complete HaloPlex gene panel was 99.5%. The mean coverage for each gene is displayed in S1 Table. Of the 290 investigated stillbirth cases, 35 (12.1%) had one (n = 31) or two (n = 4) variants with evidence supporting pathogenicity, *i*.*e*. LoF variants (nonsense, frameshift, splice site substitutions), evidence from functional studies, or previous identification of the variants in affected individuals (Table 2). The proportion of individuals harboring the same variants in ExAC NFE and SweGen was significantly lower, 4.8% (p<0.001) and 5.1% (p<0.001), respectively (Table 3). Twenty cases had a variant in a channelopathy gene *(i*.*e*. *CACNB2*, *GPD1L*, *KCNH2*, *KCNJ8*, *KCNQ1*, *RYR2*, *SCN5A* and *TRPM4)*, whereas 15 cases had one (n = 11) or two (n = 1) variants in cardiomyopathy genes *(i*.*e*. *ABCC9*, *BAG3*, *DES*, *DSG2*, *DSP*, *MYBPC3*, *NEBL*, *NEXN*, *TNNI3* and *TTN)*. Three cases (34, 286 and 290) had one variant in a cardiomyopathy gene and one in a channelopathy gene *(CSRP3* and *TRPM4*, *PKP2* and *ANK2*, *MYH7* and *KCNH2*, respectively). The proportion of individuals harboring putative pathogenic variants in the different categories are displayed in Table 3. As *KCNQ1* was the gene in which most putative pathogenic SNVs were identified in the stillbirth cohort (n = 5), all missense and LoF variants recorded in ExAC NFE were systematically searched for in ClinVar, to get an approximation of how common pathogenic SNVs are in the European population for this gene. The results showed a significantly higher total number of observations of putative pathogenic alleles in *KCNQ1* in relation to wild type alleles in the study cohort compared with ExAC NFE (5/580 (0.86%) vs. 219/66 740 (0.33%), p = 0.046). A Monte Carlo permutation test with 10 000 iterations was performed to compare the proportion of pathogenic SNVs in the stillbirth cohort to ExAC NFE across all 70 genes included in the gene panel. Groups of 50 variants from each cohort were drawn in each iteration. The average proportions of putative pathogenic SNVs did not differ between the stillbirth cohort and ExAC NFE, which were calculated as 3.1% and 3.2%, respectively.

**Table 2:** Putative pathogenic SNVs in 290 stillbirth cases.

Gene	Case	Sex	GA	Diagnostic findings	Nucleotide change (genomic level, genome build GRCh37/hg19)	Nucleotide change (cDNA level)	Amino acid change	dbSNP ID	Scaled CADD score	MAF (ExAC NFE)	MAF (SweGen)	ACMG class^1^	Evidence supporting pathogenicity
*ABCC9*	112	XX	32+3	Incomplete lung lobation	chr12:22063090C>T	c.1320+1G>A^hom^	-	rs139620148	27.6	0.000060	0.0015	3	Splice site substitution. Likely skip of exon 8.
*ANK2*	286	XY	41+1	N/A	chr4:114294462C>T	c.11716C>T	p.(Arg3906Trp)	rs121912706	35	0.001724	0.0030	3	Identified in LQTS patients. Functional evidence.^2^
*BAG3*	82	XY	31+0	N/A	chr10:121431911C>T	c.652C>T	p.(Arg218Trp)	rs397514506	32	0.000030	-	3	Identified in DCM patients. Functional evidence.^2^
*CACNB2*	69	XX	39+4	Umbilical cord complication	chr10:18828181C>T	c.1439C>T	p.(Thr480Ile)	rs143326262	22.2	0.002388	-	3	Identified in BrS patients.^2^
*CSRP3*	34	XX	27+4	N/A	chr11:19207878C>T	c.299G>A	p.(Arg100His)	rs138218523	24.9	0.002008	0.0050	3	Identified in HCM patients.^2^
*DES*	82	XY	31+0	N/A	chr2:220285586G>A	c.934G>A	p.(Asp312Asn)	rs34337334	34	0.000015	-	3	Identified in DCM patients. Functional evidence.^2^
*DSG2*	217	XX	24+0	-	chr18:29125783G>A	c.2434G>A	p.(Gly812Ser)	rs121913010	28.6	0.000030	-	3	Identified in ARVC patients. Functional evidence.^2^
	250	XY	23+3	-	chr18:29126689C>T	c.3340C>T	p.(Gln1114*)	-	42	-	-	4	Creates a premature STOP codon, not described previously.
*DSP*	101	XY	39+0	N/A	chr6:7580494G>C	c.4071G>C	p.(Glu1357Asp)	rs569786610	23.6	-	-	3	Identified in cases of sudden cardiac death.^2^
	22	XX	40+3	Inward rotation of feet and lower legs	chr6:7584376C>G	c.6881C>G	p.(Ala2294Gly)	rs147000526	28.8	0.000974	0.0005	3	Identified in DCM patients.^2^
*GPD1L*	149	XY	35+6	-	chr3:32181723A>G	c.370A>G	p.(Ile124Val)	rs72552293	3.81	0.002397	0.0040	3	Identified in SIDS cases and BrS patients. Functional evidence.^2^
	164	XY	35+6	IUGR									
	238	XY	37+6	Large heart and liver, hyperlobation of right lung, overlapping toes									
	273	XX	38+4	-									
*KCNH2*	290	XY	36+1	N/A	chr7:150645550G>A	c.2674C>T	p.(Arg892Cys)	rs201627778	32	0.000215	-	3	Identified in cases of sudden cardiac death.^2^
*KCNJ8*	11	XY	23+0	-	chr12:21918667G>A	c.1265C>T	p.(Ser422Leu)	rs72554071	16.5	0.002518	0.0005	3	Identified in sudden death cases and BrS patients. Functional evidence.^2^
*KCNQ1*	5	XY	27+0	-	chr11:2466656G>A	c.328G>A	p.(Val110Ile)	rs199472677	22.7	0.000145	-	3	Identified in LQTS patients. Functional evidence.^2^
	228	XY	37+4	N/A									
	63	XY	29+5	-	chr11:2594115A>G	c.820A>G	p.(Ile274Val)	rs199472728	25.1	0.000365	0.0005	3	Identified in SIDS cases. Functional evidence.^2^
	108	XX	27+4	Accessory spleen	chr11:2608860C>T	c.1189C>T	p.(Arg397Trp)	rs199472776	33	0.000270	-	3	Identified in a stillbirth case and in LQTS patients. Functional evidence.^2^
	88	XY	Full term	Renal agenesis	chr11:2610069G>A	c.1378G>A	p.(Gly460Ser)	rs199472783	10.07	0.000047	-	3	Identified in a case of SIDS and in LQTS patients.^2^
*MYBPC3*	61	XY	26+0	Incomplete lung lobation, IUGR	chr11:47359047C>T	c.2497G>A	p.(Ala833Thr)	rs199865688	32	0.002730	0.0030	3	Identified in a case of SIDS and in DCM patients.^2^
	202	XX	37+1	N/A									
*MYH7*	290	XY	36+1	N/A	chr14:23894566C>T	c.2348G>A	p.(Arg783His)	rs397516142	25.2	0.000030	-	3	Identified in HCM patients.^2^
*NEBL*	256	XX	36+4	Accessory spleen	chr10:21157673C>T	c.604G>A	p.(Gly202Arg)	rs137973321	19.89	0.003120	0.0020	3	Identified in DCM patients. Functional evidence.^2^
*NEXN*	46	XY	29+0	-	chr1:78395131A>C	c.995A>C	p.(Glu332Ala)	rs201763096	23.2	0.002821	0.0005	3	Identified in HCM patients.^2^
*PKP2*	286	XY	25+2	N/A	chr12:32949101G>T	c.2431C>A	p.(Arg811Ser)	rs139734328	34	0.001154	0.0015	3	Identified in ARVC patients.^2^
*RYR2*	150	XX	36+3	N/A	chr1:237934127G>A	c.11497G>A	p.(Asp3833Asn)	-	25.2	0.000104	-	3	Identified in CPVT patients.^2^
	245	XX	34+5	N/A	chr1:237774125C>T	c.4747C>T	p.(Pro1583Ser)	rs200070226	32	0.000131	-	3	Identified in ARVC patients.^2^
	4	XX	37+1	N/A	chr1:237791277G>A	c.6337G>A	p.(Val2113Met)	rs186906598	19.5	0.000752	0.0015	3	Identified in a case of sudden unexplained death.^2^
*SCN5A*	209	XY	22+2	N/A	chr3:38645430C>A	c.1663G>T	p.(Glu555*)	-	24.2	-	-	4	Creates a premature STOP codon, not described previously.
	55	XY	28+4	Hydrops fetalis, talipes equinovarus	chr3:38645235G>A	c.1858C>T	p.(Arg620Cys)	rs199473577	25.1	-	-	3	Identified in BrS patients.^2^
	177	XX	24+2	N/A	chr3:38592513C>T	c.5350G>A	p.(Glu1784Lys)	-	33	-	-	3	Identified in BrS and LQTS patients.^2^
*TNNI3*	199	XY	25+3	-	chr19:55666189G>A	c.292C>T	p.(Arg98*)	-	40	0.000151	-	3	Creates a premature STOP codon.
*TRPM4*	104	XY	30	-	chr19:49684650_49684657del	c.1195_1202del	p.(Leu399Glyfs*11)	-	34	0.000030	-	4	Frameshift, creates a premature STOP codon 10 codons downstream.
	34	XX	27+4	N/A	chr19:49703651A>T	c.2740A>T	p.(Lys914*)	rs140799936	54	0.002213	0.0020	3	Creates a premature STOP codon. Identified in BrS patients. Functional evidence.^2^
	261	XY	27+4	Lung hypoplasia									
	26	XY	24	-	chr19:49713558T>C	c.3224T>C	p.(Leu1075Pro)	rs144421653	28.5	0.000195	-	3	Identified in BrS patients. Functional evidence.^2^
*TTN*	65	XY	37+4	-	chr2:179581821C>A	c.25639+1G>T	-	-	24.9	-	-	4	Splice site substitution. Likely skip of exon 89. Not described previously.

GA: gestational age, CADD: Combined Annotation Dependent Depletion, MAF: minor allele frequency, ARVC: arrhythmogenic right ventricular cardiomyopathy, BrS: Brugada syndrome, CHD: congenital heart defects, CPVT: catecholaminergic polymorphic ventricular tachycardia, DCM: dilated cardiomyopathy, FAF: familial atrial fibrillation, HCM: hypertrophic cardiomyopathy, IUGR: intrauterine growth restriction, LQTS: long QT syndrome, N/A: not available, SIDS: sudden infant death syndrome. Underlined cases occur twice in the table. ^hom^ = homozygous variant.

^1^ = Clasification according to ACMG guidelines, where 3 = variant of unknown significance (VUS) and 4 = likely pathogenic. Specific criteria used for each classification are displayed in S3 Table.

^2^ = Variant specific references are displayed in S3 Table.

**Table 3:** Proportions of individuals with pathogenic SNVs in the study cohort, compared with the corresponding proportions of the same variants in ExAC NFE and SweGen.

Category	Putative pathogenic SNVs, study cohort	Putative pathogenic SNVs, ExAC NFE*	P-Value, study cohort vs. ExAC NFE (Fisher’s exact test)	Putative pathogenic SNVs, SweGen*	P-Value, study cohort vs. SweGen (Fisher’s exact test)
All genes	35/290 (12.07%)	1,776/33 370 (5.32%)	<0.001	51/1 000 (5.10%)	<0.001
Channelopathy genes	23/290 (7.93%)	977/33 370 (2.70%)	<0.001	23/1 000 (2.30%)	<0.001
Cardiomyopathy genes	15/290 (5.17%)	972/33 370 (2.72%)	0.015	28/1 000 (2.80%)	0.061

* = Only SNVs identified in the study cohort are included

**Fig 1:**
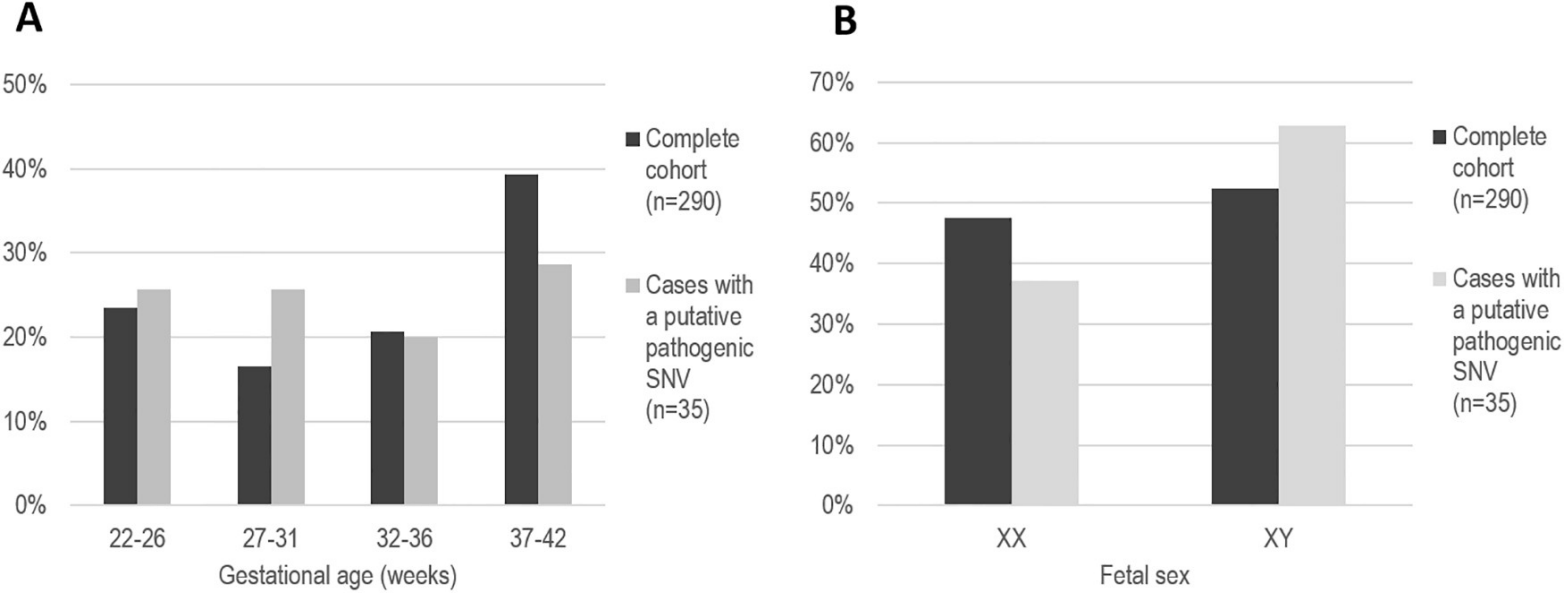
Gestational age (A) and sex (B) distribution of the stillbirth cases.

We found no support for a difference between stillbirth cases with putative pathogenic SNVs compared with the complete cohort in regard to gestational age (p = 0.429) and sex distribution (p = 0.284) (Fig 1). In addition to the variants with evidence supporting pathogenicity, 144 missense variants of unknown significance were identified in 110 stillbirth cases (S2 Table).

## Discussion

We have analyzed DNA from 290 stillbirth cases for prevalence of pathogenic SNVs in 70 genes associated with heart disease. To our knowledge, this is the first MPS-based study including a large stillbirth cohort, and according to our results, SNVs with evidence supporting pathogenicity was identified in as many as 12.1% of the cases. The proportion was significantly higher than the corresponding proportion for the same variants in ExAC NFE, (5.3%, p<0.001) as well as in SweGen (5.1%, p<0.001). When divided into the different disease categories, *i*.*e*. channelopathies and cardiomyopathies, the significant difference was seen only for channelopathies (Table 3).

Previous studies have mainly focused on stillbirth in association with LQTS. Crotti *et al* studied 91 stillbirth cases for SNVs in the most common LQTS susceptibility genes, *i*.*e*. *KCNQ1*, *KCNH2* and *SCN5A*, and identified three putative pathogenic variants (3.3%) [8]. The proportion of putative pathogenic SNVs for the same genes in our study was 3.1% (n = 9), *i*.*e*. very similar to what was reported by Crotti. One variant, *KCNQ1*, p.(Arg397Trp), was identified in both studies. Crotti *et al* showed that this variant caused a significant reduction in current densities across the potassium channel encoded by *KCNQ1*, compared with the wild type channel [8]. Furthermore, the total number of pathogenic alleles in *KCNQ1* observed in our cohort was significantly higher compared with ExAC NFE (p = 0.046), which supports that SNVs in this gene might play a role in stillbirth. In addition to the most common LQTS genes, one case in our cohort harbored a putative pathogenic SNVs in *ANK2*. Taken together, LQTS associated SNVs were identified in 10 cases (3.4%) of the stillbirth cases included in the present study.

Three cases (1%) harbored putative pathogenic SNVs in *RYR2*, associated with CPVT. CPVT is one of the most severe cardiac channelopathies, and is characterized by ventricular arrhythmias causing syncope, cardiac arrest and sudden cardiac death, predominantly in young patients including infants [11]. Thereby, these SNVs are good candidates for being associated with stillbirth. BrS associated SNVs were identified in 5.2% (n = 15) of the cases in the cohort. As BrS has mainly been described in association with sudden death in adults, its role in stillbirth is difficult to interpret. However, one of the BrS associated SNVs are worth highlighting, namely *GPD1L*, p.(Ile124Val). This variant was identified in four cases, and has been associated with sudden infant death syndrome (SIDS) in a previous study [24]. According to ExAC NFE, it has a minor allele frequency (MAF) of 0.24% in the European population, whereas the MAF in our cohort is 0.69%, *i*.*e*. almost three times as high (p = 0.054). Although not statistically significant, this might indicate that this variant is a risk factor for stillbirth as well as SIDS.

In our cohort, SNVs with evidence supporting pathogenicity in genes associated with cardiomyopathies (HCM, DCM and ARVC) were identified in 12 cases. As cardiomyopathies are progressive disorders which are generally not detected during the early years of life, they have not been studied in association with stillbirth previously. However, increasing evidence suggests that they might play a role in SIDS [13,25]. Brion *et al* studied 286 SIDS cases for variants in genes associated with HCM and found variants with possibly damaging effects in 4% of the cases [13]. One of their identified SNVs, *MYBPC3*, p.(Ala833Thr), was detected in two of our cases. Brion *et al* hypothesized that their identified variants might cause sudden cardiac death even in the absence of a cardiac phenotype, but they do also emphasize the possibility that the variants could be non-disease causing rare variants [13]. Furthermore, the proportion of putative pathogenic variants associated with cardiomyopathies identified in this study was not significantly higher than the corresponding proportion in ExAC NFE and SweGen. The Monte Carlo permutation test revealed no significant difference in proportions of putative pathogenic variants between the study cohort and ExAC NFE, which probably reflects that the majority of the 70 genes included in the panel are indeed not associated with stillbirth.

Although the putative pathogenic SNVs identified in this study had a significantly higher prevalence in in the stillbirth cohort compared with ExAC NFE data, the results should be interpreted with caution. As the cohort included in this study is substantially smaller than the one included in ExAC NFE, there is a high probability that MAFs of rare alleles are overestimated, and do not reflect the true MAFs in all cases. Nonetheless, to our knowledge, this is the largest cohort of stillbirth cases investigated for pathogenic SNVs in a large set of genes associated with heart disease that has been analyzed to date. Thereby it provides some insight to the frequency of putative pathogenic SNVs in stillbirth cases. However, there are additional limitations to the study which need to be addressed. No parental DNA samples were available, and hence it is unknown whether the identified variants are inherited or of *de novo* origin. This information would otherwise have provided additional support for or against a clinical significance of the variants. Additionally, no clinical information regarding the parents was available, and therefore it is unknown whether there is a history of heart disease or recurrent stillbirth in any of the families. Furthermore, we did not perform any functional studies on the identified variants. Because of the inherent limitations of the study, most of the putative pathogenic variants identified in this study only qualify as variants of unknown significance (VUS) when classified in accordance to the current state-of-the-art guidelines used in a clinical setting, formulated by ACMG [21]. Hence, the SNVs displayed in Table 2 should not be interpreted as verified pathogenic variants that, without further investigation, could be used for carrier testing and/or prenatal testing in a clinical laboratory. Although we have based our classifications on damage prediction of the variants and previously published data, studies suggest that pathogenicity of several reported LQTS and cardiomyopathy associated variants is overestimated [26–28]. Indeed, recent data reveals that the importance of several cardiomyopathy- and BrS related genes—some of which are included in our gene panel—is probably not as high as previously thought [29,30]. Conversely, some of the 144 missense variants of unknown significance displayed in S2 Table might be associated with heart disease, but have not yet been reported as such. Additional research is required to further clarify the clinical impact of the SNVs identified in this study.

Better knowledge of the etiology of stillbirth is needed in order to achieve a reduction in the stillbirth rate. Our results give further support to the hypothesis that cardiac channelopathies might contribute to stillbirth. Screening for pathogenic SNVs in genes associated with heart disease might be valuable in cases of stillbirth where today’s conventional investigation does not reveal the underlying cause of fetal demise.

## Supporting information

S1 Table Mean sequencing coverage of the 70 genes included in the HaloPlex gene panel. (XLSX)

S2 Table Identified SNVs of unknown clinical significance in 290 stillbirth cases. (XLSX)

S3 Table Variant specific references and ACMG classification. (DOCX)

S4 Table Complete, raw dataset. (XLSX)
