# Supplementary material for: Identification of putative pathogenic single nucleotide variants (SNVs) in genes associated with heart disease in 290 cases of stillbirth
Source: PLoS One. 2019 Jan 7;14(1):e0210017. doi: 10.1371/journal.pone.0210017 (PMC6322759; doi:10.1371/journal.pone.0210017)
Supplement: S3 Table — (DOCX) [file pone.0210017.s003.docx]

***Supplementary table 3.* Variant specific references and ACMG classification.**

| **Gene** | **Nucleotide change (genomic level, genome build GRCh37)** | **Nucleotide change (cDNA level)** | **Amino acid change** | **Evidence supporting pathogenicity** | **ACMG criteria** | **Reference(s)** |
| --- | --- | --- | --- | --- | --- | --- |
| *ABCC9* | chr12:22063090C>T | c.1320+1G>A^hom^ | - | Splice site substitution. Likely skip of exon 8. | PVS1  PP3 | **-** |
| *ANK2* | chr4:114294462C>T | c.11716C>T | p.(Arg3906Trp) | Identified in LQTS patients. Functional evidence. | PS3  PP3  PP5 | Mohler PJ, Splawski I, Napolitano C et al: A cardiac arrhythmia syndrome caused by loss of ankyrin-B function. Proceedings of the National Academy of Sciences of the United States of America 2004; 101: 9137-9142. |
| *BAG3* | chr10:121431911C>T | c.652C>T | p.(Arg218Trp) | Identified in DCM patients. Functional evidence. | PS3  PP3  PP5 | Arimura T, Ishikawa T, Nunoda S, Kawai S, Kimura A: Dilated cardiomyopathy-associated BAG3 mutations impair Z-disc assembly and enhance sensitivity to apoptosis in cardiomyocytes. Human mutation 2011; 32: 1481-1491. |
| *CACNB2* | chr10:18828181C>T | c.1439C>T | p.(Thr480Ile) | Identified in BrS patients. | PP3  PP5 | Burashnikov E, Pfeiffer R, Barajas-Martinez H et al: Mutations in the cardiac L-type calcium channel associated with inherited J-wave syndromes and sudden cardiac death. Heart rhythm : the official journal of the Heart Rhythm Society 2010; 7: 1872-1882. |
| *CSRP3* | chr11:19207878C>T | c.299G>A | p.(Arg100His) | Identified in HCM patients. | PP3  PP5 | Ng D, Johnston JJ, Teer JK et al: Interpreting secondary cardiac disease variants in an exome cohort. Circulation Cardiovascular genetics 2013; 6: 337-346.  Andersen PS, Havndrup O, Hougs L et al: Diagnostic yield, interpretation, and clinical utility of mutation screening of sarcomere encoding genes in Danish hypertrophic cardiomyopathy patients and relatives. Human mutation 2009; 30: 363-370. |
| *DES* | chr2:220285586G>A | c.934G>A | p.(Asp312Asn) | Identified in DCM patients. Functional evidence. | PS3  PP3  PP5 | Taylor MR, Slavov D, Ku L et al: Prevalence of desmin mutations in dilated cardiomyopathy. Circulation 2007; 115: 1244-1251. |
| *DSG2* | chr18:29125783G>A | c.2434G>A | p.(Gly812Ser) | Identified in ARVC patients. Functional evidence. | PS3  PP3  PP5 | Gehmlich K, Asimaki A, Cahill TJ et al: Novel missense mutations in exon 15 of desmoglein-2: role of the intracellular cadherin segment in arrhythmogenic right ventricular cardiomyopathy? Heart rhythm : the official journal of the Heart Rhythm Society 2010; 7: 1446-1453. |
|  | chr18:29126689C>T | c.3340C>T | p.(Gln1114*) | Creates a premature STOP codon, not described previously. | PVS1  PM2  PP3 | - |
| *DSP* | chr6:7580494G>C | c.4071G>C | p.(Glu1357Asp) | Identified in cases of sudden cardiac death. | PM2  PP3  PP5 | Zhao Q, Chen Y, Peng L et al: Identification of rare variants of DSP gene in sudden unexplained nocturnal death syndrome in the southern Chinese Han population. International journal of legal medicine 2016; 130: 317-322. |
|  | chr6:7584376C>G | c.6881C>G | p.(Ala2294Gly) | Identified in DCM patients. | PP3  PP5 | Ng D, Johnston JJ, Teer JK et al: Interpreting secondary cardiac disease variants in an exome cohort. Circulation Cardiovascular genetics 2013; 6: 337-346. |
| *GPD1L* | chr3:32181723A>G | c.370A>G | p.(Ile124Val) | Identified in SIDS cases and BrS patients. Functional evidence. | PS3  PP5 | Van Norstrand DW, Valdivia CR, Tester DJ et al: Molecular and functional characterization of novel glycerol-3-phosphate dehydrogenase 1 like gene (GPD1-L) mutations in sudden infant death syndrome. Circulation 2007; 116: 2253-2259.  Hedley PL, Jorgensen P, Schlamowitz S et al: The genetic basis of Brugada syndrome: a mutation update. Human mutation 2009; 30: 1256-1266. |
| *KCNH2* | chr7:150645550G>A | c.2674C>T | p.(Arg892Cys) | Identified in cases of sudden cardiac death. | PP3  PP5 | Ng D, Johnston JJ, Teer JK *et al*: Interpreting secondary cardiac disease variants in an exome cohort. *Circulation Cardiovascular genetics* 2013; **6:** 337-346.  Campuzano O, Sanchez-Molero O, Allegue C et al: Post-mortem genetic analysis in juvenile cases of sudden cardiac death. Forensic science international 2014; 245: 30-37. |
| *KCNJ8* | chr12:21918667G>A | c.1265C>T | p.(Ser422Leu) | Identified in sudden death cases and BrS patients. Functional evidence. | PS3  PP5 | Haissaguerre M, Chatel S, Sacher F et al: Ventricular fibrillation with prominent early repolarization associated with a rare variant of KCNJ8/KATP channel. Journal of cardiovascular electrophysiology 2009; 20: 93-98.  Medeiros-Domingo A, Tan BH, Crotti L et al: Gain-of-function mutation S422L in the KCNJ8-encoded cardiac K(ATP) channel Kir6.1 as a pathogenic substrate for J-wave syndromes. Heart rhythm : the official journal of the Heart Rhythm Society 2010; 7: 1466-1471.  Barajas-Martinez H, Hu D, Ferrer T et al: Molecular genetic and functional association of Brugada and early repolarization syndromes with S422L missense mutation in KCNJ8. Heart rhythm : the official journal of the Heart Rhythm Society 2012; 9: 548-555. |
| *KCNQ1* | chr11:2466656G>A | c.328G>A | p.(Val110Ile) | Identified in LQTS patients. Functional evidence. | PS3  PP3  PP5 | Cordeiro JM, Perez GJ, Schmitt N et al: Overlapping LQT1 and LQT2 phenotype in a patient with long QT syndrome associated with loss-of-function variations in KCNQ1 and KCNH2. Canadian journal of physiology and pharmacology 2010; 88: 1181-1190. |
|  | chr11:2594115A>G | c.820A>G | p.(Ile274Val) | Identified in SIDS cases. Functional evidence. | PS3  PP3  PP5 | Arnestad M, Crotti L, Rognum TO et al: Prevalence of long-QT syndrome gene variants in sudden infant death syndrome. Circulation 2007; 115: 361-367.  Rhodes TE, Abraham RL, Welch RC et al: Cardiac potassium channel dysfunction in sudden infant death syndrome. Journal of molecular and cellular cardiology 2008; 44: 571-581. |
|  | chr11:2608860C>T | c.1189C>T | p.(Arg397Trp) | Identified in a stillbirth case and in LQTS patients. Functional evidence. | PS3  PP3  PP5 | Crotti L, Tester DJ, White WM et al: Long QT syndrome-associated mutations in intrauterine fetal death. Jama 2013; 309: 1473-1482.  Moss AJ, Shimizu W, Wilde AA et al: Clinical aspects of type-1 long-QT syndrome by location, coding type, and biophysical function of mutations involving the KCNQ1 gene. Circulation 2007; 115: 2481-2489. |
|  | chr11:2610069G>A | c.1378G>A | p.(Gly460Ser) | Identified in a case of SIDS and in LQTS patients. | PP3  PP5 | Arnestad M, Crotti L, Rognum TO et al: Prevalence of long-QT syndrome gene variants in sudden infant death syndrome. Circulation 2007; 115: 361-367.  Kapplinger JD, Tester DJ, Salisbury BA et al: Spectrum and prevalence of mutations from the first 2,500 consecutive unrelated patients referred for the FAMILION long QT syndrome genetic test. Heart rhythm : the official journal of the Heart Rhythm Society 2009; 6: 1297-1303. |
| *MYBPC3* | chr11:47359047C>T | c.2497G>A | p.(Ala833Thr) | Identified in a case of SIDS and in DCM patients. | PP3  PP5 | Brion M, Allegue C, Santori M et al: Sarcomeric gene mutations in sudden infant death syndrome (SIDS). Forensic science international 2012; 219: 278-281.  Hershberger RE, Norton N, Morales A, Li D, Siegfried JD, Gonzalez-Quintana J: Coding sequence rare variants identified in MYBPC3, MYH6, TPM1, TNNC1, and TNNI3 from 312 patients with familial or idiopathic dilated cardiomyopathy. Circulation Cardiovascular genetics 2010; 3: 155-161. |
| *MYH7* | chr14:23894566C>T | c.2348G>A | p.(Arg783His) | Identified in HCM patients. | PP3  PP5 | Waldmuller S, Muller M, Rackebrandt K et al: Array-based resequencing assay for mutations causing hypertrophic cardiomyopathy. Clinical chemistry 2008; 54: 682-687. |
| *NEBL* | chr10:21157673C>T | c.604G>A | p.(Gly202Arg) | Identified in DCM patients. Functional evidence. | PS3  PP5 | Purevjav E, Varela J, Morgado M et al: Nebulette mutations are associated with dilated cardiomyopathy and endocardial fibroelastosis. Journal of the American College of Cardiology 2010; 56: 1493-1502.  Maiellaro-Rafferty K, Wansapura JP, Mendsaikhan U et al: Altered regional cardiac wall mechanics are associated with differential cardiomyocyte calcium handling due to nebulette mutations in preclinical inherited dilated cardiomyopathy. Journal of molecular and cellular cardiology 2013; 60: 151-160. |
| *NEXN* | chr1:78395131A>C | c.995A>C | p.(Glu332Ala) | Identified in HCM patients. | PP3  PP5 | Wilson KD, Shen P, Fung E et al: A Rapid, High-Quality, Cost-Effective, Comprehensive and Expandable Targeted Next-Generation Sequencing Assay for Inherited Heart Diseases. Circulation research 2015; 117: 603-611. |
| *PKP2* | chr12:32949101G>T | c.2431C>A | p.(Arg811Ser) | Identified in ARVC patients. | PP3 PP5 | Fressart V, Duthoit G, Donal E et al: Desmosomal gene analysis in arrhythmogenic right ventricular dysplasia/cardiomyopathy: spectrum of mutations and clinical impact in practice. Europace : European pacing, arrhythmias, and cardiac electrophysiology : journal of the working groups on cardiac pacing, arrhythmias, and cardiac cellular electrophysiology of the European Society of Cardiology 2010; 12: 861-868.  Klauke B, Kossmann S, Gaertner A et al: De novo desmin-mutation N116S is associated with arrhythmogenic right ventricular cardiomyopathy. Human molecular genetics 2010; 19: 4595-4607. |
| *RYR2* | chr1:237774125C>T | c.4747C>T | p.(Pro1583Ser) | Identified in ARVC patients. | PP3 PP5 | Roux-Buisson N, Gandjbakhch E, Donal E et al: Prevalence and significance of rare RYR2 variants in arrhythmogenic right ventricular cardiomyopathy/dysplasia: results of a systematic screening. Heart rhythm : the official journal of the Heart Rhythm Society 2014; 11: 1999-2009. |
|  | chr1:237791277G>A | c.6337G>A | p.(Val2113Met) | Identified in a case of sudden unexplained death. | PP3  PP5 | Tester DJ, Medeiros-Domingo A, Will ML, Haglund CM, Ackerman MJ: Cardiac channel molecular autopsy: insights from 173 consecutive cases of autopsy-negative sudden unexplained death referred for postmortem genetic testing. Mayo Clinic proceedings 2012; 87: 524-539. |
|  | chr1:237934127G>A | c.11497G>A | p.(Asp3833Asn) | Identified in CPVT patients. | PP5 | Adler A, Sadek MM, Chan AY et al: Patient Outcomes From a Specialized Inherited Arrhythmia Clinic. Circulation Arrhythmia and electrophysiology 2016; 9: e003440. |
| *SCN5A* | chr3:38645430C>A | c.1663G>T | p.(Glu555*) | Creates a premature STOP codon, not described previously. | PVS1  PM2  PP3 | - |
|  | chr3:38645235G>A | c.1858C>T | p.(Arg620Cys) | Identified in BrS patients. | PM2  PP3 PP5 | Kapplinger JD, Tester DJ, Alders M et al: An international compendium of mutations in the SCN5A-encoded cardiac sodium channel in patients referred for Brugada syndrome genetic testing. Heart rhythm : the official journal of the Heart Rhythm Society 2010; 7: 33-46. |
|  | chr3:38592513C>T | c.5350G>A | p.(Glu1784Lys) | Identified in BrS and LQTS patients. | PM2  PP3 PP5 | Makita N, Behr E, Shimizu W et al: The E1784K mutation in SCN5A is associated with mixed clinical phenotype of type 3 long QT syndrome. The Journal of clinical investigation 2008; 118: 2219-2229.  Shim SH, Ito M, Maher T, Milunsky A: Gene sequencing in neonates and infants with the long QT syndrome. Genetic testing 2005; 9: 281-284. |
| *TNNI3* | chr19:55666189G>A | c.292C>T | p.(Arg98*) | Creates a premature STOP codon. | PVS1  PP3 | - |
| *TRPM4* | chr19:49684650_49684657del | c.1195_1202del | p.(Leu399Glyfs*11) | Frameshift, creates a premature STOP codon 10 codons downstream. | PVS1  PM2  PP3 | - |
|  | chr19:49703651A>T | c.2740A>T | p.(Lys914*) | Creates a premature STOP codon. Identified in BrS patients. Functional evidence. | PS3  PP3 PP5 | Liu H, Chatel S, Simard C et al: Molecular genetics and functional anomalies in a series of 248 Brugada cases with 11 mutations in the TRPM4 channel. PloS one 2013; 8: e54131. |
|  | chr19:49713558T>C | c.3224T>C | p.(Leu1075Pro) | Identified in BrS patients. Functional evidence. | PS3  PP3 PP5 | Liu H, Chatel S, Simard C et al: Molecular genetics and functional anomalies in a series of 248 Brugada cases with 11 mutations in the TRPM4 channel. PloS one 2013; 8: e54131. |
| *TTN* | chr2:179581821C>A | c.25639+1G>T | - | Splice site substitution. Likely skip of exon 89. Not described previously. | PVS1  PM2  PP3 | - |
